# A continuation-dynamic constitution analysis approach based on digital stable marker tracing and study on simulation of ecological tidal water diversion

**DOI:** 10.1038/s41598-023-39611-7

**Published:** 2023-12-28

**Authors:** Mengya Xing, Simin Qu, Hui Xu, Peng Shi, Xing Chen, Feifei Ji, Minton Liu

**Affiliations:** College of Hydrology and Water Resources, Hohai University, Nanjing, 210098 China

**Keywords:** Hydrology, Pollution remediation, Fluid dynamics

## Abstract

Water Diversion Projects have become increasingly popular in improving water quality in various water ecosystems. However, these projects also require a more comprehensive evaluation. In this study, we introduced a digital stable marker tracing module and proposed a continuation-dynamic constitution analysis approach. We applied this approach to analyze the ecological tidal water diversion in Changshu town, China. The results showed that the mean diversion water age of the Yangtze River water source was 10.80 h, the residence time of the background water source in Baimaotang was approximately 4.0 h, and the contribution of inflow water sources from tributaries accounted for 15% of discharges. The results can demonstrate practicality of our approach in quantitatively evaluating water diversion impacts and optimizing cooperative diversion projects. Furthermore, our discussion led to the design of an ecological tidal water diversion based on optimized cooperative diversion, which showed element-complementary and whole-comprehensive effects. This indicates that the ecological tidal water diversion can extend the impact of cooperative diversion. The continuation-dynamic constitution analysis approach enhances the tracing capacity of inflow constitution and enables the distinction of different time-varying distributions of each inflow constitution. Therefore, this approach holds promise as an embedded “Digital stable marker tracing” module in the model.

## Introduction

Ecological tidal water diversion has become a standard engineering measure and a significant focus of research in the field of ecological tidal water diversions. This is because tidal river networks typically have abundant water resources, and the dynamic nature of tides makes them suitable for hydrodynamic water diversion. However, the frequent and complex reversing tidal currents pose scientific challenges to cooperative water diversion. Since the 1990s, there has been an increasing implementation of ecological tidal water diversion aimed at improving water eco-environment and river health^1^. These projects aim to supply the deficient environmental factors to the water ecosystem through water diversion and other necessary measures such as pollution interception and ecological restoration^2–5^. Depending on the specific characteristics of the water ecosystem, ecological tidal water diversions typically targets include: (i) improving ecological carrying capacity, e.g., Emergency Water Transmission Project of Lower Tarim River and Zalong Wetland Emergency Water Supply Project; (ii) increasing water environment capacity, e.g., Pearl River Estuary Pressure and Salt Repair Emergency Water Diversion, Water Diversion from the Yangtze River to Lake Taihu project.

In the Taihu Lake Basin, which is characterized by urban river networks and lake marshes, the water quality is commonly described as low-carbon and high-nitrogen. The average carbon–nitrogen ratio is typically below the critical threshold value of 2.86, indicating a deficiency in self-net carbon–nitrogen ratio^6^. As a result, since the first water diversion test through Wangyu River in 2002, more cities, including Changshu town, have been implementing ecological water supplying projects along the water diversion lines^7–12^. In-depth diversion researches on effect evaluations and scientific optimizations are generally increasing^13–16^; however, it is still necessary to study on the scientific analysis approach to accurately describe and optimize the ecological tidal water diversion plans.

Hence, we propose a continuation-dynamic constitution analysis approach based on digital stable marker tracing. We applied this approach to analyze and optimize cooperative diversion in the center river network of Changshu town, using the tidal hydrodynamic forces of the Yangtze River and Wangyu River to introduce water resources and improve the water environment. Additionally, we designed an ecological tidal water diversion that considers water pollution control and ecological restoration to extend the effectiveness of cooperative diversion.

The objectives of this study are: (i) Based on digital stable marker tracing to propose the continuation-dynamic constitution analysis approach; (ii) apply continuation-dynamic constitution analysis to describe and optimize the cooperative diversion in Changshu tidal river network, China; (iii) further discuss the implementation effects and characteristics of ecological tidal water diversion in Changshu tidal river network, China.

## Results

### Water diversion design

#### Study area

It is located in the center of Changshu town, 31° 35ʹ–31° 46ʹ N, 120° 40ʹ–121° 03ʹ E, with a surface area of 60 km^2^ around; and the ground elevation is mostly 3–7 m, Woosung datum, with “Northwest-high, Southeast-low” topographic characteristic.

**Figure 1:**
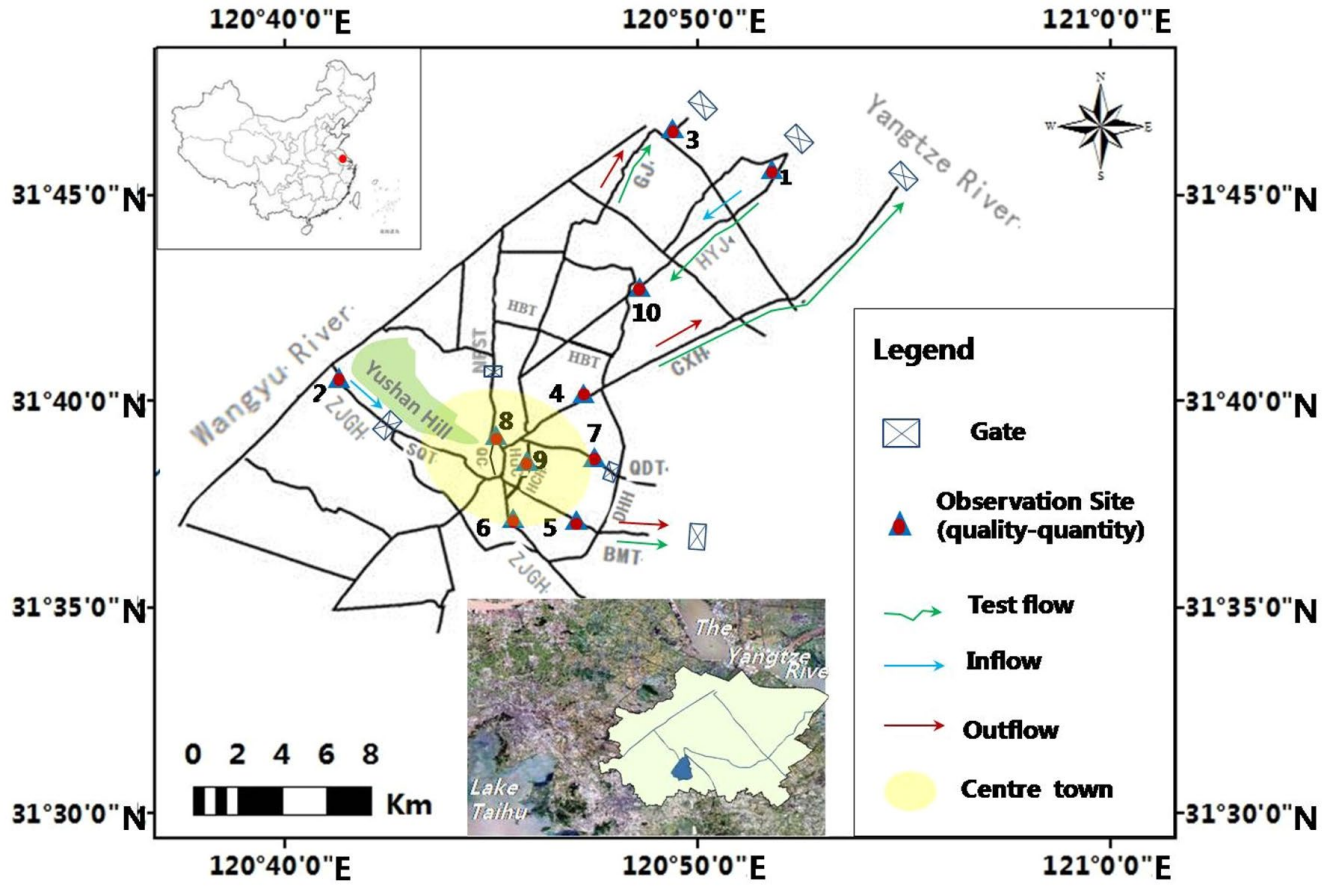
Map of the study area. The 2 *diversion* channels with *gates* are Haiyangjing (HYJ), Zhangjiaganghe (ZJGH); the 3 *drainage* channels with *gates* are Gengjing (GJ), Changxuhe (CXH), Baimaotang (BMT). In addition, there are 9 main rivers marked out: the 4 entering the centre town as Qinchuan (QC), Huchenghe (HuC), Shanqiantang (SQT) and Hunchenghe (HCH); the 2 *gates* controlled as Nanfushantang (NFST) and Qingduntang (QDT); and the other 3 as Huabantang (HBT), Hengjingtang (HJT) and Donghuanhe (DHH). The map was created using QGIS (QGIS Development Team, software version 3.16.13, https://www.qgis.org) and the satellite image was from 91weitu (91weitu Development Team, software version 19.3.4, https://www.91weitu.com).

This area mainly contains 14 rivers and 7 gates, showed in Fig. 1: the 5 channels as Haiyangjing, Zhangjiaganghe, Gengjing, Changxuhe and Baimaotang; the 4 rivers entering the center town as Qinchuan, Huchenghe, Shanqiantang and Hunchenghe; the 5 rivers near the center town as Huabantang, Nanfushantang, Qingduntang, Hengjingtang and Donghuanhe; and the 7 gates include: the 5 estuary gates between the Yangtze River and Haiyangjing, Zhangjiaganghe, Gengjing, Changxuhe, Baimaotang, respectively; the 2 internal gates on Nanfushantang, Qingduntang.

#### Water diversion

As the reversing tidal current and local human activities, the study river network exhibits low environmental capacity and weak self-purification capacity^17–19^. Therefore, Chen et al. scholars designed a prototype water diversion test to transfer water resources by tidal dynamic from the Yangtze River to the center river network on May 14th, 2014^20^. The water transfer routes and quantity–quality observation sections are shown in Fig. 1; the measured processes on selected Sections are shown in Fig. 7.

Based on the prototype water diversion test’s suggestion that: “extend the water diversion time and increase the diversion channels”, authors designed two water diversion projects to improve the ecological water diversion effect of the tidal river network; see Table 1 and Fig. 1. Design1 aimed to extend the water diversion time and lengthened the water diversion duration from 13.5 to 36 h. Design2 aimed to increase the diversion channel and selected a new track: Wangyu River-Zhangjiaganghe-center river network to introduce clean water in 24 h.

**Table 1:** Prototype test and Simulation designs of water diversion.

Name	Water diversion channels	Duration	Water drainage channels	Gates operation	
				Diversion control	Drainage control
Test	The Yangtze River-HYJ-center river network	13.5 h	CXH-the Yangtze River and BMT-the Yangtze River and GJ-the Yangtze River	HYJ gate	CXH gate and BMT gate and GJ gate
Design1	The Yangtze River-HYJ-center river network	36 h		HYJ gate	
Design2	Wangyu River-ZJGH-center river network	24 h		ZJGH gate	

Design1 aimed to extend the water diversion time and lengthen the water diversion duration of the Yangtze River-HYJ-center river network channel from 13.5 to 36 h.

Design2 aimed to increase the number of diversion channels and select channel Wangyu River-ZJGH-center river network, and introduce clean water for 24 h.

The gates that showed in Table 1 and Fig. 1 have operation rules as follows: (i) when the tide Level increases to the ordinary water Level of 3.2 m, open the diversion channels gates: Haiyangjing gate, Zhangjiagang gate, and keep the drainage channels gates closed; (ii) when tide Level decreases to the ordinary water Level of 3.2 m, close the diversion channels gates and open the drainage channel gates: Gengjing gate, Changxuhe gate and Baimaotang gate, to control flow discharge and keep water quantity balance of local river network; (iii) keep both the Nanfushantang gate and Qingduntang gate closed during the whole water diversion.

#### Results assessment

**Figure 2:**
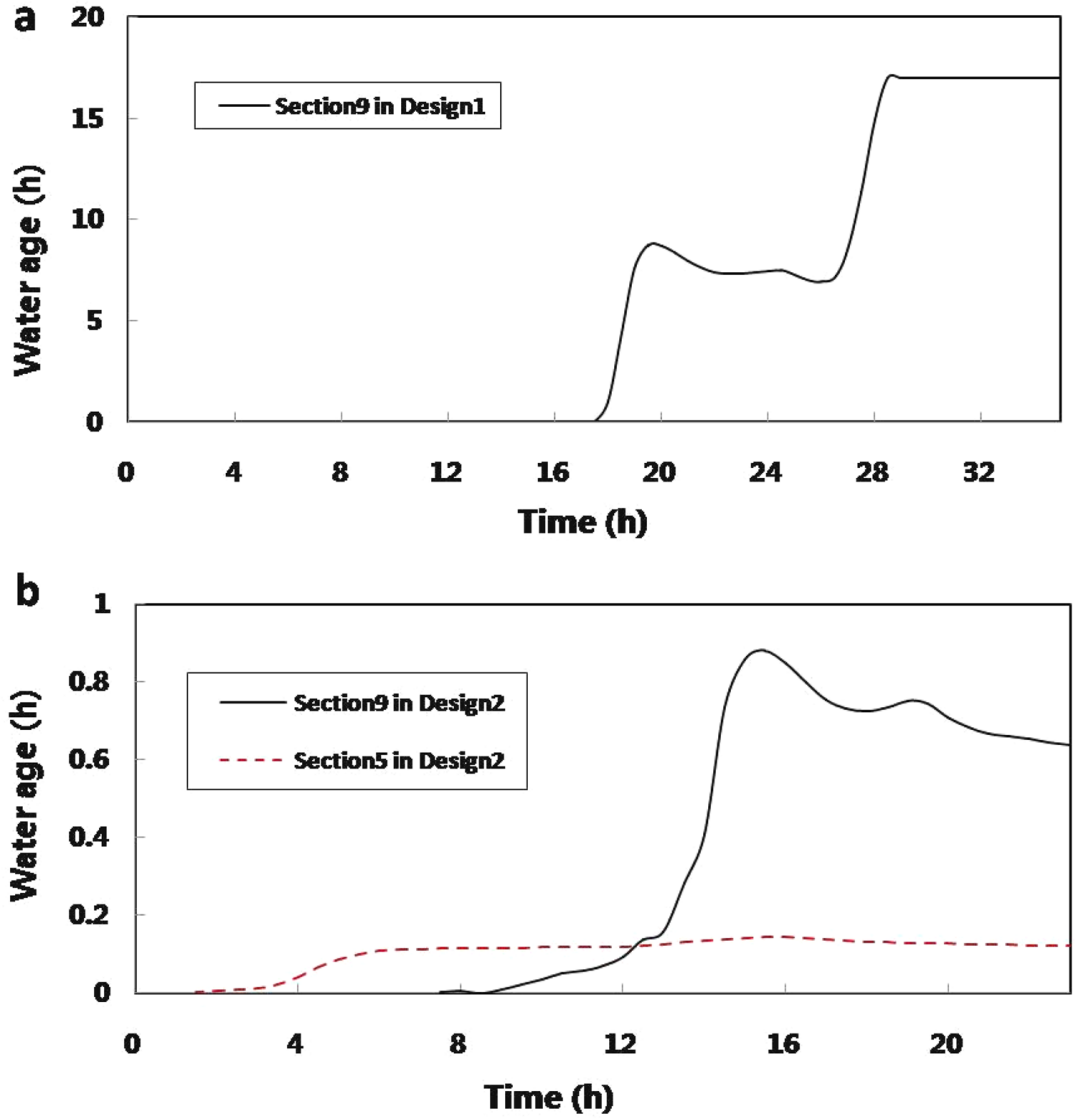
Time series of water ages on Section5 and Section9 in designed WDPs. (**a**) The assessment sections in designed WDP1; (**b**) the assessment sections in designed WDP2.

To evaluate the water diversion results of Design1 and Design2 (Details in Table 1), two assessment sections were selected: Section9 and Section5 (see Fig. 2). Section9 is located in the center of the tidal river network, which is the main water import area with complex flow conditions. Section5 is located downstream in the southeast of town, where a large proportion of the tidal river network flow dumps in.

### Water age and residence time analysis

In Design1, see Fig. 2a, the peak discharge at Section9 was 11.9 m^3^/s, the residence time of the background water source in Huanchenghe was about 2.0 h, and the mean water age of the diversion water source from Yangtze River at Section9 was 10.80 h. In comparison, the peak discharge at Section5 was only 3.70 m^3^/s, and the residence time of the background water source in Baimaotang was about 4.0 h. No Yangtze River water source flowed into Section5 at the end of the simulation.

In Design2, see Fig. 2b, the peak discharge at Section5 was 8.15 m^3^/s, the residence time of the background water source in Baimaotang was about 9.0 h, and the mean water age of the diversion water source from Wangyu River at Section5 was only 0.11 h. Meanwhile, the peak discharge at Section9 was only 4.16 m^3^/s, the residence time of the background water source in Huanchenghe was also 9.0 h, and the mean water age of the diversion water source from Wangyu River at Section9 was only 0.45 h.

It can be inferred that the WDP Design1 from the Yangtze River had abundant water resources and topographic advantages that went into the center of town. Both helped lengthen the water age of water diversion source in Design1 to the tidal river network. For a clean and abundant water diversion, the long water age is significant to improve the effect of ecological water supplements. Some previous studies have also found that residence time was positively associated to the nutrients^17,21^, which means the inflow water resource of Design1 could significantly affect the nutrients in background water. However, with the increased flow path distance, drainage channels’ response in southern river networks to supplement is limited, as the ecological water resource has not yet arrived there in simulation. Some previous studies have also found that the clean supplement may not involve the entire water area^22,23^.

As for the WDP Design2 from Wangyu River, benefitting from the location of diversion channel Zhangjiaganghe close to the center of town, the water diversion source of Design2 can arrive at Section5 in 2 h and improve the water environment capacity of the drainage river networks. However, due to the limitation of ecological supplement discharge, inflow in diversion channel Zhangjiaganghe has low velocity and short water age. Since long-term trickle usually has a positive effect on reducing the nutrient concentration^24,25^, the long-time diversion processes in this situation may be advantaged.

### Continuation-dynamic constitution analysis

#### Assessment Section9

**Figure 3:**
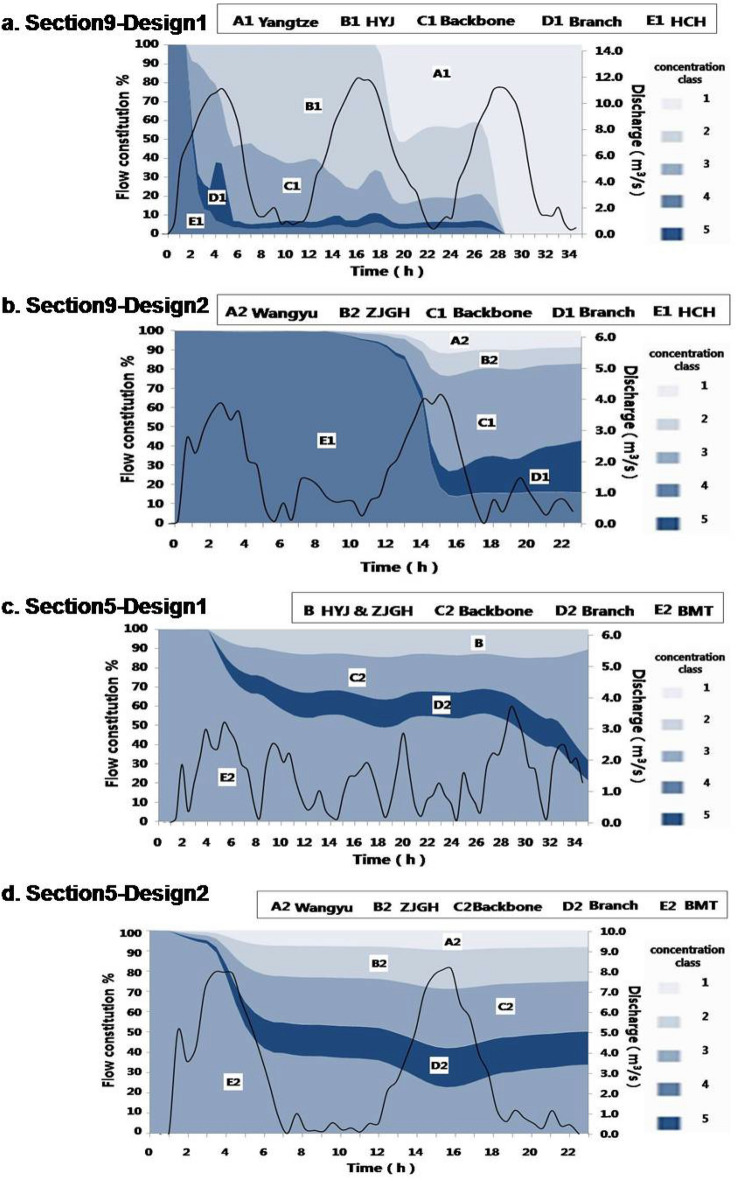
Time-variant quality-quantity processes at assessment sections in designed WDP1 and WDP2. (**a**) Blue color gradients represent different water-quality Level s, the letter in every blue block corresponds the inflow water constitution name. (**b**) Horizontal axis: as a timeline, to indicate the water diversion process from start time 0 to end time n. (**c**) Left vertical axis: as a percentage axis, to reflect the proportion of inflow water constitution at different times, fit with the blue blocks in different depths. (**d**) Right vertical axis: as a flow axis, to represent the inflow on section at different times, fit with the black curve.

Water quality-quantity continuation-dynamic components of Section9 on Huanchenghe in the center of town in water diversion Design1 are shown in Fig. 3a. In the initial 6 h, the mean water environment quality Level in the assessed section stayed in Level 3 to Level 5. During 6 to 17 h, as diversion water became the dominant source, the background water source of Haiyangjing in Level 2 accounted for 50% of inflow and improved the water quality at Section9. From 17 h, the water source of the Yangtze River in Level 1 started reaching the section continuously. And during 17 to 28 h, the water diversion channel Haiyangjing and water diversion source from the Yangtze River accounted for about 80% of inflow into Section9, which effectively increased the water environment capacity. After 28 h, the Level 1 water diversion source from the Yangtze River accounted for 100% inflow. We can infer that: (i) after 17 h of water diversion, the water environment capacity in the tidal river network effectively increased. Moreover, the water quality at Section9 achieved the target Grade IV after about 28 h; (ii) water supplement in this situation was compelling that the diversion water source of Yangtze River accounted for 33.1% during the whole diversion on average.

The results of water diversion Design2 of Section9 of Huanchenghe in the center of town are shown in Fig. 3b. In the first 12 h, the contribution of the background water source of Huanchenghe in Level 4 was steadily above 90%; then, the inflow water source increased continuously. After 16 h, flow proportions from Wangyu River, Zhangjiaganghe and background water source of Huanchenghe became stable at about 10%, 10% and 15%, respectively; meanwhile, inflow from backbone rivers in Level 3 and branch rivers in Level 5 accounted for 65%. However, the discharge of backbones roughly reduced by 15% eventually, the same as the increased contribution of branches. The above analysis suggests that: (i) since the topographic effect that “Northwest-high, Southeast-low” of the center of town, there were a large proportion of discharges from major town river networks draining into Section9; (ii) the water quality was mostly influenced by the tributaries so that in the final 8 h, the environment quality of this area deteriorated mildly; (iii) water supplement in this situation was minimal that the diversion water source of Wangyu River only accounted for 4.13% during the whole diversion in average.

#### Assessment Section5

The water quality-quantity continuation-dynamic components of Section5 on the drainage channel Baimaotang in water diversion Design1 are shown in Fig. 3c. Inflow water constitutions at Section5 remained stable in the first 5 h. The contribution of background water sources of Haiyangjing and Zhangjiaganghe in Level 2 became stable at about 15% of discharges. The background water source in Baimaotang and the inflow water source from backbone Rivers accounted for 50%, and 20%, respectively, and both of their water qualities were in Level 3. Inflow water sources from tributaries had the worst water quality as Level 5, and their contribution accounted for 15% of discharges. No inflow water resource from the Yangtze River reached here during the whole diversion. Besides, during 12 to 28 h, the mean discharge at Section5 was only 0.54 m^3^/s due to the reversing action of water flow. After 28h, the proportion of discharges from the northern river networks increased, which might be affected by topographic factors. In a word: (i) in this situation, the diversion distance from the source Yangtze River was too long to supply the drainage channel Baimaotang ecologically; (ii) river networks around Baimaotang outside the south of town did not get significant ecological water supplement.

The results of water diversion Design2 at Section5 on the drainage channel Baimaotang are shown in Fig. 3d. Since the short distances of the flow path, the diversion water source of Wangyu River reached Section5 in the first 2 h and inflow water constitutions at Section5 became stable after 6 h. The contribution of background water resources and backbone rivers’ inflow water resources in Level 3 accounted for more than 50% of discharges; the better quality inflow water source in Level 1 and Level 2 accounted for about 20–25%; and the proportion of inflow tributaries in Level 5 accounted for about 15–20%. On average, the diversion water source from Wangyu River accounted for 6.75% during the whole diversion, which nearly did not help improve the environment. We infer that: (i) ecological water supplement in this situation was stable but not apparent to the drainage channel Baimaotang and surrounding river networks; (ii) the water quality of Baimaotang and surrounding river networks could improve a little after this WDP and the inflow water source wouldn’t have negative impacts on water quality of Secion5.

### Optimization of the cooperative diversion

The WDP management methods are crucial in improving diversion efficiency^26^. According to the water diversion effect of designed WDPs and the results of continuation-dynamic constitution analysis, we optimize the cooperative diversion.

On the one hand, the river networks outside the south of town mainly undertake the outflow from the center of town because of the “Northwest-high, Southeast-low” topography characteristics. On the other hand, to avoid the water-Level jacking and reversing of the flow of several central reaches, which are caused by two diversion sources, we optimize the diversion scheme. Firstly, supply the ecological water mainly from the Yangtze River to the tidal river network; secondly, when the flow in center river network becomes stable, introduce water resources from the Wangyu River to consolidate the effect of environmental water supplement for the river network outside the south of town.

## Discussion

To keep the enduring effect of cooperative diversion, we designed a comprehensive diversion scheme, including measures as: pollution interception, cooperative water diversion and ecological restoration, to discuss the impact of ecological tidal water diversion application (Table 2).

**Figure 4:**
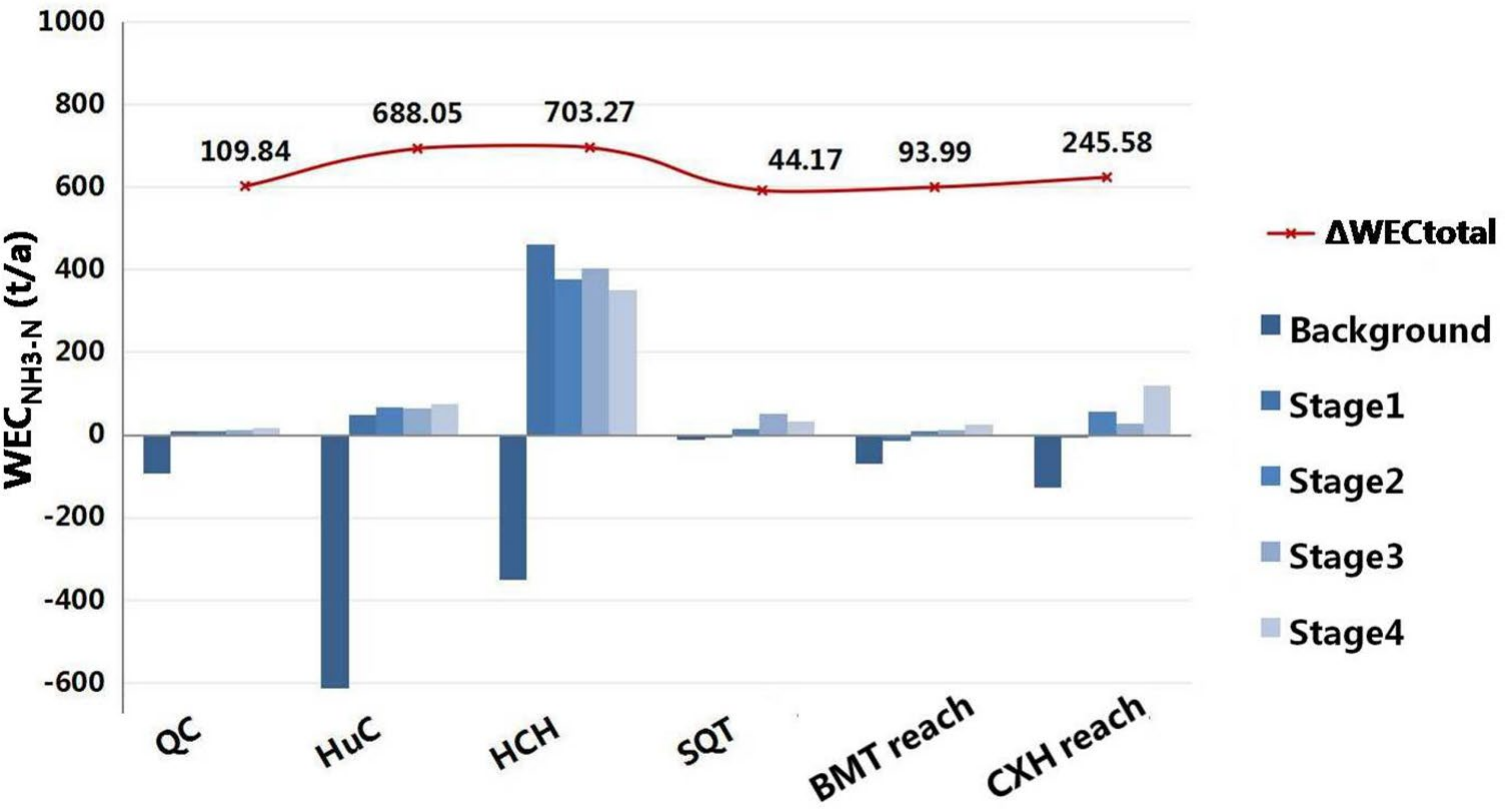
Water environment capacity of channels in central river network.

Based on the simulation of the Ecological Water Diversion Project scheme in Table 2, we calculated the water environment capacity of the tidal river network in different stages, and the results were showed in Fig. 4. In the first stage, due to the effect of pollution interception, ecological restoration and especially the abundant and clean water diversion from the Yangtze River and Haiyangjing channel, the rest environment capacity of the central river network had increased from − 1760.38 to 497.61 t/a. This huge improvement of the rest of WEC is the sum of main rivers’ WEC in the center of town (see Fig. 4). The significant impact of the Yangtze River water diversion on study river network could be confirmed by the previous water diversion tests^20,27^. During the second stage, many rivers’ water qualities were continuously improved except Huanchenghe, which was affected by drainage flow from the center of town. In the third stage, the addition of a water source from Wangyu River through the Zhangjiaganghe gate significantly increased the water environment capacity of River Shanqiantang, a channel directly connected with Zhangjiaganghe, from 13.31 t/a to the maximum value of 50.87 t/a. At the end of the simulation, the total water environmental capacity of NH_3_-N reached 588.81 t/a, and the water quality of the main channels in the center of town met the target. The Ecological Water Diversion Project proved to be effective in maintaining the enduring effect of cooperative diversion.

**Table 2:**
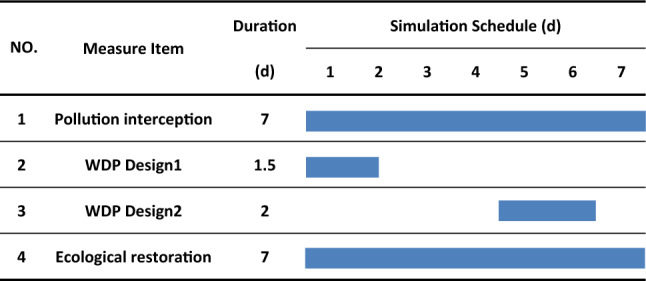
Gantt Chart for Changshu Ecological Water Diversion Project timelines.

**Figure 5:**
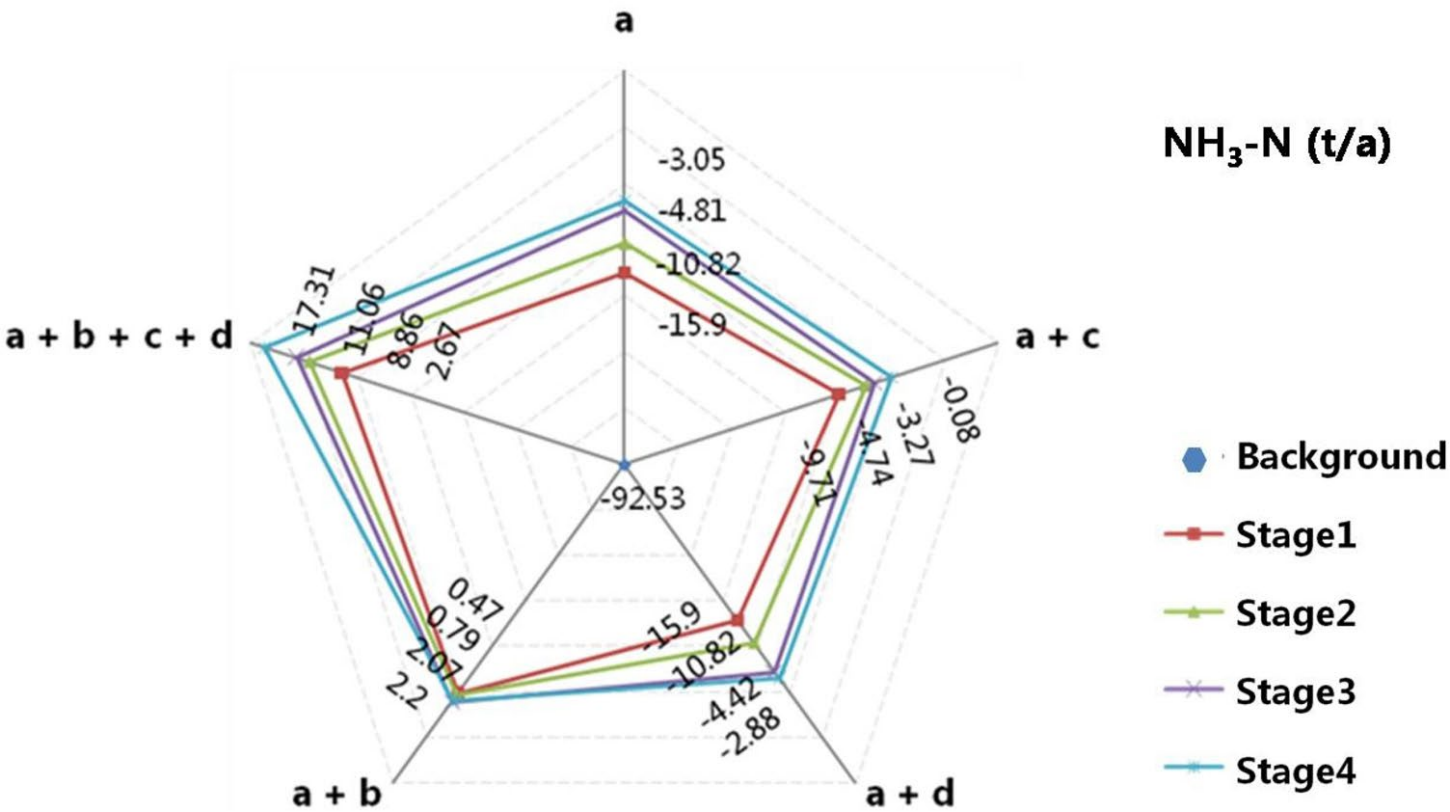
Water environment capacity of River QC in different stages. a. Pollution interceptions of rivers include Rivers Qinchuan (QC), Huchenghe (HuC) and Nanfushantang (NFST); b. ecological restoration as urban scenic river; c. ecological water diversion through Haiyangjing (HYJ); d. ecological water diversion through Zhangjiaganghe (ZJGH).

Taking the River Qinchuan located in the central business district of Changshu, see Fig. 5, as an instance. The structure of this river, with a closed gate upstream connected to Beifushantang, passing through the center of town from north to south, and downstream connected to Zhangjiaganghe, resulted in limited water inflow. Previous studies suggested that the closed gate could reduce the river’s self-purification capacity due to slow flow velocity^13^. In the simulation, various measures were implemented, including: a. Pollution interceptions of rivers include Rivers Qinchuan, Huchenghe and Nanfushantang; b. ecological restoration as an urban scenic river; c. ecological water diversion through Haiyangjing; d. ecological water diversion through Zhangjiaganghe. Effects of these mentioned measures were operated in single and combined, and the water environmental capacity of River Qinchuan in different simulating stages was shown in Fig. 5.

The combined effect of all measures proved to be the most effective, with positive capacities at the end of Day7, while the capacities in measures “a+c” and “a+d” were still negative. It highlighted the element-complementary effect of measures in the Ecological Water Diversion Project. Moreover, the concentration of NH_3_-N in pollution interception measure “a” significantly reduced, and the water environment capacity in measure “a+b” preliminarily became positive. Both can prove the importance of pollution interception and ecological restoration in improving the self-purification ability of river channels^28^. Similarly, there has also been proposed that 75% of the international river pollution interception research was devoted to the restoration of river ecology, and about 35% was trying to restore the lost riparian vegetation and wetland community^29^.

Comparing the simulation results of different measures in Fig. 5, it was found that the Ecological Water Diversion Project had a better improvement and restoration effect than the sum of four single measures, see Formula (1). It reflected the comprehensive effect of the Ecological Water Diversion Project, i.e., the coupling of project elements and their synergistic impact, where “1 + 1 > 2”.

1 $$\left|a+b+c+d\right|-\left|a+c\right|-\left|a+d\right|-\left|a+b\right|+\left|a\right|\times 2=12.1>0.$$

## Methods

**Figure 6:**
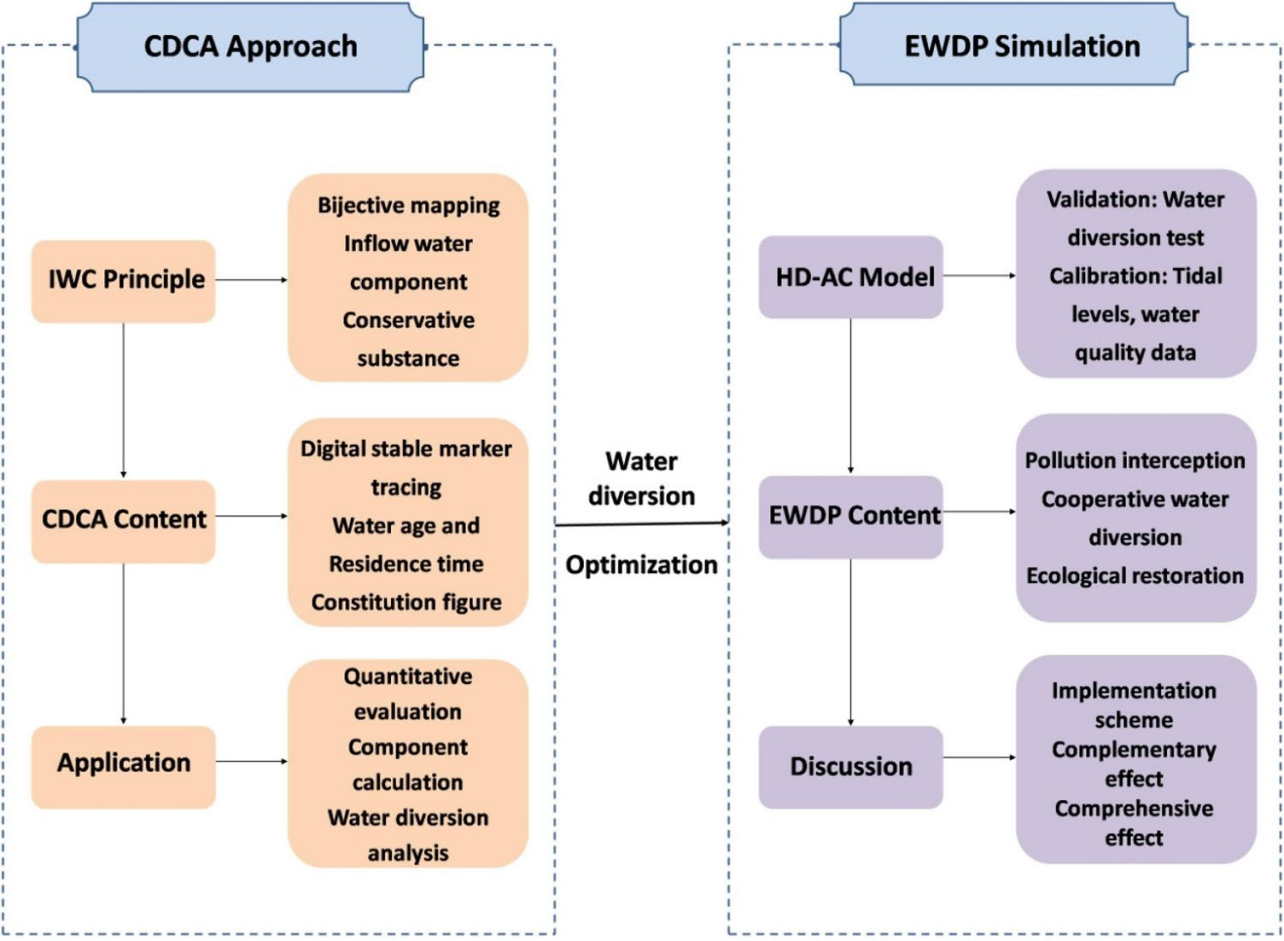
The flow chart of methodologies used in this study.

The methodologies schematic diagram of this study is shown in Fig. 6. Firstly, a coupled hydrodynamic-water quality model was constructed to simulate the hydrodynamic distribution and the water quality variation along the river during water diversion processes. Secondly, the model was calibrated by the monitored data of estuary-tidal Level s and monitored water-quality data from June to August 2014, and validated by the observed data of the prototype water diversion test. Thirdly, the optimization of designed water diversion projects, see Table 1 and Fig. 1, was simulated by hydrodynamic-aquatic model and analyzed by continuation-dynamic constitution analysis. Lastly, the optimized result of the designed water diversion as a cooperative diversion project was implemented into an Ecological Water Diversion Project, which effects were discussed in water environment capacity.

### Digital stable marker tracing

#### Principle of inflow water constitution

Let X be the set of all inflow water components, Y be the set of Conservative Substance, such that F: X → Y is a bijective mapping.

Conservative Substance refers to the mass that only has a physical effect, for example, transport, dilution and mixing, no chemical reaction or material loss while moving with the flow. That means they meet the mass conservation law, and their substance attenuation coefficient is zero.

Suppose *C*_*i,j*_(0,0) is the concentration of Conservative Substance *j* ($j \in Y$) contained in the inflow water component *i* ($i \in X$), at the initial spatial and temporal coordinates, then get Formula (2):

2 $$C_{i,j} (0,0) = \left\{ {\begin{array}{*{20}c} 1 & {X(i) \to Y(j),} \\ 0 & {Otherwise.} \\ \end{array} } \right.$$

where *C*_*i,j*_(0,0) is non-dimensional.

#### Digital stable marker tracing labels

Particle tracking is helpful in understanding the process of water movement^30,31^. In our simulation, the particle tracking was realized by Digital Tracing Labels:

Firstly, establish the bijective mapping with Conservative Substance and all inflow water constitutions of inflow rivers of the assessment sections. Secondly, label every inflow water constitution by Conservative Substance in the model. Then, simulate the time-variant concentration of each Conservative Substance according to the percentages of all water sources that flow into one section simultaneously. At last, the partition of each inflow water component can be quantified at the assessment sections.

### Continuation-dynamic constitution analysis

#### Water age and residence time

In order to explore the hydraulic characteristic of flows with different concentrations, we calculated the residence time of background water source^32–35^ and the mean Water age of diversion water source on assessment section^33,36,37^, according to the theory of Water Age and Residence Time^38,39^.

Residence time of background water source means the required time for background water source particle that first leaving the assessment section during water diversion.

Age of diversion source water on assessment sections means the required time for water particle that transporting from the source to assessment section. Formula (3) showed the Mean age concentration of particle *a*_*i*_ per Principle of inflow water constitution.

3 $${a}_{i}=\frac{1}{n}\sum_{t=1}^{n}{\tau }_{i}{C}_{i,j}\left(t,{x}_{0}\right),$$

where *τ*_*i*_ denotes the age of diversion water source particle *i* on the assessment section; *C*_*i,j*_*(t,x*_*0*_*)* denotes the concentration of Conservative Substance *j* contained in the inflow water component *i*, at the temporal coordinate *t* and the assessment-section spatial coordinate* x*_0_. *n* denotes the maximum diversion time.

#### Continuation-dynamic constitution figure

The continuation-dynamic constitution figure shows the variation of every inflow water constitution in different water-quality concentration Level s on the assessment sections during the diversion.

### Hydrodynamic: water quality model

#### Hydrodynamic model

The one-dimensional-unsteady hydrodynamic model describes the water flow state of a river or estuary based on the vertically-integrated mass and momentum conservation equation, showed as Formula (4) and (5), respectively, that is, the one-dimensional-unsteady Saint–Venant equation. The equations can be written as follows^40^.

4 $$\frac{\partial A}{{\partial t}} + \frac{\partial Q}{{\partial x}} = q$$

5 $$\frac{\partial Q}{{\partial t}} + \frac{{\partial \, \left( {\alpha \frac{{Q^{2} }}{A}} \right)}}{\partial x} + gA\frac{{\partial {\text{ h}}}}{\partial x} + \frac{{{\text{g}}Q\left| Q \right|}}{{ARC^{2} }} = 0$$

where *A* is the area of cross sections (m^2^), *x* and *t* denote the spatial and temporal coordinates, respectively; *Q* and *q* denote the discharge (m^3^/s) and lateral inflow (m^3^/s), respectively; *α* is the momentum correction factor, *h* is the mean water depth of cross section (m), *R* is the hydraulic radius,* C* is the Chezy coefficient, and *g* is the gravitational acceleration.

#### Water quality model

The water quality model is a one-dimensional advection dispersion model, and describes the water quality variation in channels based on 1-D convection–diffusion equation, see Formula (6). In order to assess the status of water quality during the diversion, the representative index NH_3_-N was used to reflect the influence of human beings in the center of the town^24,41^.

6 $$\frac{\partial AI}{{\partial t}} + \frac{\partial QI}{{\partial x}} - \frac{\partial }{\partial x}\left( {AD\frac{\partial I}{{\partial x}}} \right) = - AKI + I_{2} q$$

where *I*, *D* and* K* denote the NH_3_-N concentration (mg/L), diffusion coefficient (m^2^/s) and linear attenuation coefficient (d^−1^) of a water quality state variable, respectively; *I*_2_ is concentration of point source or confluence (mg/L).

#### Model parameters

The monitored data of estuary-tidal Level s and water qualities from June to August 2014 were used for parameter calibration, and the observed data of the prototype water diversion test^20^ were used for validation. The results are as follows:

(i) The monitored discharge on Sections of water diversion channels was used as the upper boundary; (ii) the corresponding monitored estuary-tidal Level on exports of drainage channels was adopted as the lower boundary; (iii) the closed boundary was set as the ordinary water Level 3.2 m; (iv) the range of river channel roughness coefficients *n* were 0.025–0.032; (v) the range of NH_3_-N daily attenuation coefficients *K* were 0.08/d–0.2/d.

**Figure 7:**
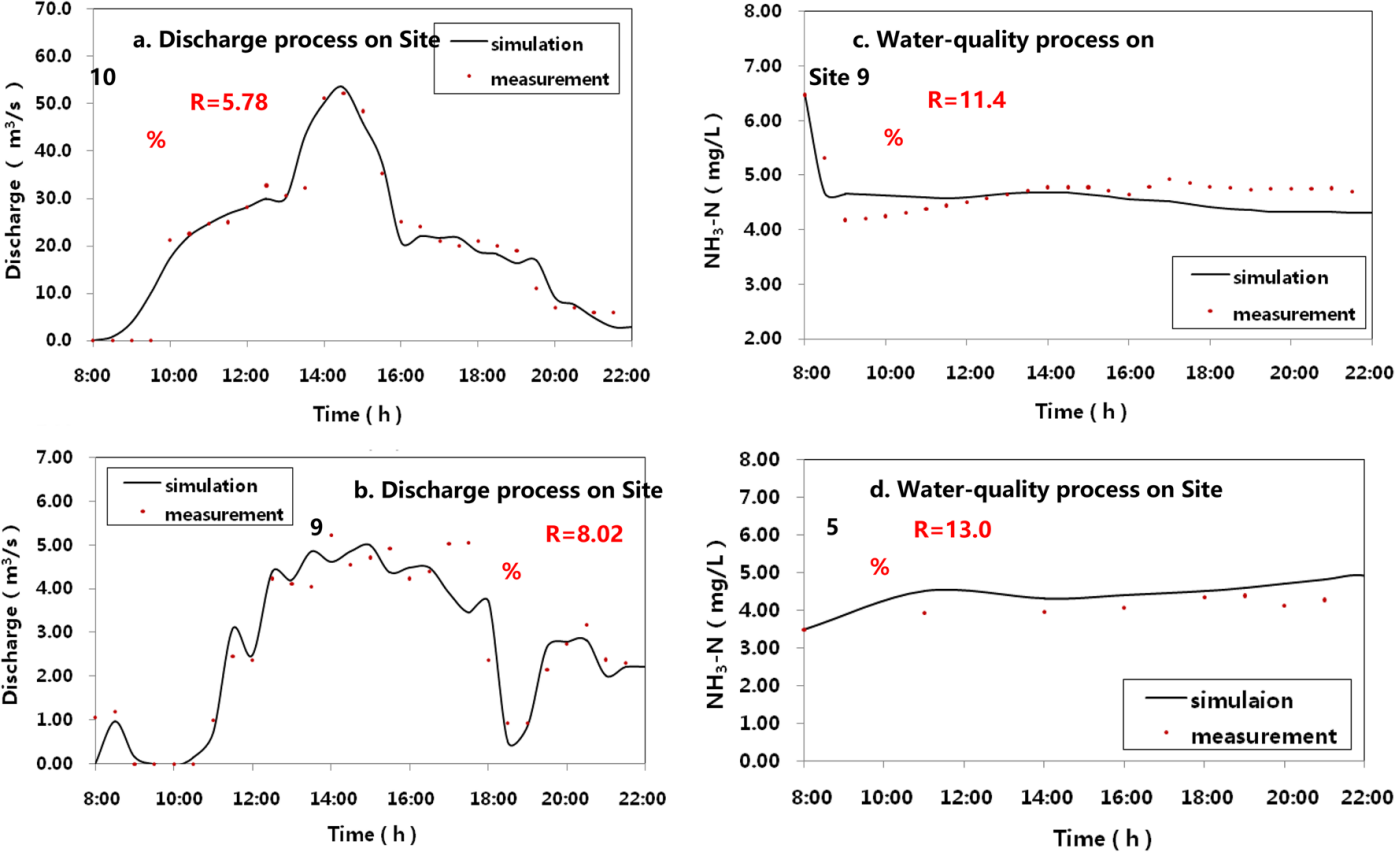
Results of hydrodynamic-water quality model on Section. The results of advection dispersion simulation were marked as curves and the measured processes on selected Sections were marked in points. *R* denotes the relative error between the simulated and measured data.

The model performance is shown in Fig. 7. The relative errors between measurements and simulations were 15% for discharge and 20% for NH_3_-N concentration. Excellent model performance ensured further application for studying the designed diversion projects simulation.

### Ecological Water Diversion Project

#### Pollution interception

Controlling pollutant loads of point sources is an essential part in water environment management, especially in urban areas. Based on the water-quality situation and requirement and characteristic of research area water environment that: urban domestic sewage as a significant pollutant has more than half NH_3_-N load in total, while urban and farmland runoff as main non-point pollutant sources have very large majorities of COD and NH_3_-N, respectively; we set the Sewage Interception Rate as 96% for the urban domestic sewage by improving the receiving and collecting rate of domestic sewage, as 85% for the industrial enterprise pollution by improving the standard of industrial enterprise pollution discharge, and as 75% for the farmland runoff by increasing the ecological buffer zone that cut off the non-point pollution transport path.

#### Cooperative water diversion

Cooperative water diversion means supplying water factor to strengthen the hydraulic connection, further enhance the self-purification capacity and ecological corridors to restore the aquatic ecosystems^42^. We optimized the cooperative water diversion based on Table 1.

#### Ecological restoration

As the results of local aquatic investigations and hydrostatic tests of aquatic plants, we selected the common plant species that: submerged plants like goldfish algae and foxtail algae to treat TN and retrain the release of P in sediments^43^; emerged plants like reed and lotus to adsorb N, P and NH_3_-N^44^. And considering the characteristics of different types of rivers, we selected suitable aquatic plant species to build three-dimensional plant communities^45^ as following: (i) Urban scenic rivers, with slow flow velocity, commercial and residential lands along, are to set ecological floating beds. (ii) Industrial buffer rivers, with relatively slow flow velocity, industrial and domestic lands along, are to build riparian vegetation buffer zone. (iii) Navigation protective rivers, with the functions of navigation, flood control, drainage etc., industrial and agricultural lands along, are to treat bank slope in vertical sections.

#### Project scheme

Considering the cooperative diversion optimization and measures above, we propose an Ecological Water Diversion Project scheme, see Table 2. Our simulation period selected the first 7 days of significant linear decrease in water quality concentration. Previous studies confirmed that the removal rates of NH4^+^-N, TN by related aquatic plants are all above 62.3% in the first 7 days^46,47^.

## Data Availability

The data used in this paper will be made available upon request; please send a request to chenxing@hhu.edu.cn. We highly appreciate Changshu Water Affairs Bureau for the survey data of Changshu water environment.
